# Mental Health Supports for Sexual and Gender Minorities Who Experience Intimate Partner Violence and Abuse: A Systematic Review of North American Literature

**DOI:** 10.1177/15248380251355918

**Published:** 2025-09-20

**Authors:** Stefan Kurbatfinski, Katherine Maurer, Jessica Whitehead, Noah Ulicki, Lee Hodge, Martin Morris, René Peltekian, Richard Henry, Zack Marshall

**Affiliations:** 1 Department of Community Health Sciences, Cumming School of Medicine, University of Calgary, Calgary, AB, Canada; 2 Alberta Children’s Hospital Research Institute, Owerko Centre, Calgary, AB, Canada; 3 School of Social Work, McGill University, Montreal, QC, Canada; 4 Toronto Metropolitan University, Toronto, ON, Canada; 5 Dalla Lana School of Public Health, University of Toronto, Toronto, ON, Canada; 6 Schulich Library of Physical Sciences, Life Sciences and Engineering, McGill University, Montreal, QC, Canada; 7 University of Waterloo, ON, Canada; 8 Department of Medicine, McGill University, Montreal, QC, Canada; 9 Lady Davis Institute for Medical Research, Jewish General Hospital, Montreal, QC, Canada

**Keywords:** intimate partner violence, sexual and gender minorities, LGBTQ+, help seeking/reporting/disclosure, intimate partner abuse, mental health effects

## Abstract

Sexual and gender minorities (SGM) experience inequitable health outcomes due to oppressive social forces (e.g., homophobia, transphobia). For SGM experiencing intimate partner violence and abuse (IPV), help-seeking is challenged by concomitant navigation of the abusive partner and discriminatory forces toward SGM. Mental health providers are an important source of support who can provide SGM experiencing IPV with the tools and resources they need to manage or leave abusive relationships. This systematic review synthesized existing literature on the help-seeking experiences of SGM in North America who experienced IPV and accessed mental health supports. This secondary review (#CRD42020139639) from a larger study used meta-aggregative methods to identify main findings. The authors searched peer-reviewed literature from MEDLINE, Embase, PsycInfo, Scopus, CINAHL, Genderwatch, and Social Science Abstracts and assessed validity using the Joanna-Briggs Institute Checklists. Four synthesized findings were identified from studies (*N* = 34) conducted in the United States (*n* = 29) and Canada (*n* = 5): (a) components of SGM-affirming spaces, (b) characteristics of mental health provision, (c) healing journey process, and (d) community awareness and familiarity with services. Mental health providers (not including couples’ counselors) were identified as positive forms of support for SGM experiencing IPV, providing opportunities for self-development and skills to manage or leave abusive relationships. SGM-affirming spaces (e.g., use of inclusive language) increased SGM comfort in IPV discussions with mental health providers. Training and acknowledgment of SGM-specific IPV, along with client-provider rapport, further underlie effective mental health supports. Increased education and outreach would promote improved access to mental health supports.

## Introduction

Intimate partner violence and abuse (IPV) consists of various forms of violent or abusive behaviors (e.g., physical, emotional, sexual, identity, financial, religious, cultural, spiritual, legal) between two or more intimate partners (Jaffray, 2021; Kurbatfinski et al., 2024a). In addition to immediate impacts, IPV can have enduring consequences, often leading to adverse outcomes related to mental and physical health, educational attainment, employment and financial status, and impacts on other relationships (Patra et al., 2018). IPV research has focused extensively on cisgender heterosexual (cisheterosexual) relationships, only recently emphasizing the unique manifestation of IPV among Two-Spirit, lesbian, gay, bisexual, trans, queer, questioning, intersex, asexual (2SLGBTQQIA+), and other sexual and gender minorities (SGM; Henry et al., 2021). However, SGM can experience unique vulnerabilities such as gender navigation, societal gender misperceptions, bullying due to identity, transmission of human immunodeficiency virus, identity concealment, and assumptions regarding sexual roles, among others (Silveira & de Morais, 2025). Although some studies suggest that IPV rates are similar between SGM and their cisheterosexual counterparts, others report higher rates among SGM (Brown & Herman, 2015), pointing to SGM as an important group in the context of IPV.

This systematic review focuses on the interconnections between IPV, SGM, and mental health, specifically exploring help-seeking experiences with mental health services. Throughout, mental health services are defined as any form of formal support provided by mental health specialists, including psychologists, psychiatrists, counselors (couples or individual), therapists, social workers, or peer support groups (with a professional facilitator). In the next sections, we focus on (a) the links between IPV, SGM, and mental health, (b) mental health services and IPV, and (c) gaps in understanding access to mental health services for SGM who experience IPV.

### IPV, SGM, and Mental Health

Multiple studies point to the links between IPV and mental health challenges (Oram et al., 2022). SGM who experience IPV are at greater risk of health and mental health concerns (B. Miller & Irvin, 2017; E. Miller et al., 2016). For example, SGM who experience IPV report higher rates of depressive symptoms (Reuter et al., 2017; Woulfe & Goodman, 2020), anxiety symptoms (Reuter et al., 2017), symptoms of posttraumatic stress disorder (Alessi et al., 2013; Roberts et al., 2010), somatic symptoms (Scheer & Mereish, 2021), and substance use concerns (Roberts et al., 2010; Scheer & Mereish, 2021). Researchers have also explored differential mental health impacts of IPV within SGM. For example, results from one study suggested that bisexual men were more likely to experience physical and sexual violence and to report negative mental health outcomes compared to homosexual and heterosexual men (Dickerson-Amaya & Coston, 2019). In another study with trans and gender diverse participants who experienced IPV, sexual, psychological, physical, and assault with injury were positively associated with anxiety, and aside from physical abuse, associated with depression (Henry et al., 2021).

In addition to the mental health impacts of IPV, mental health and substance use concerns have been identified as underlying risk factors for both using and experiencing IPV (Basting et al., 2024; Capaldi et al., 2012). As a form of interpersonal violence, IPV occurrence is a function of psychosocial and environmental factors, often intergenerationally transmitted, including experiences of childhood abuse and neglect, parental substance use, poor parent-child attachment, and cultural norms, among others (Herbert et al., 2020; Kimmes et al., 2019; Spencer et al., 2019). For SGM, navigating cisheteronormative societal norms while experiencing oppression based on sexual orientation and/or gender identity results in increased vulnerability to mental health and substance use concerns (Jacmin-Park et al., 2022). Prevalence of mental health challenges is likely to increase among SGM who navigate intersecting oppressions, including racialized or Indigenous people, migrants, people with disabilities, and/or individuals who experience socioeconomic challenges (Crenshaw, 2017; Kimmes et al., 2019).

### The Importance of Mental Health Services in IPV Journeys

Mental health supports are typically categorized as formal or informal. This review focuses on formal help-seeking, defined as “. . . assistance from professionals who have a legitimate and recognized professional role in providing relevant advice, support, and/or treatment” (Rickwood & Thomas, 2012, p. 175). Practitioners who provide mental health services include many types of professionals, including psychologists, couples counselors, therapists, psychiatrists, social workers, and support group moderators (Sutton et al., 2021). Each of these mental health providers, despite their different training, therapeutic modalities, and roles, shares a common aim assisting individuals in their mental health.

Mental health services can play a pivotal role in how SGM experiencing IPV understand, navigate, transform, or potentially leave their relationships, given the predominant role that mental health plays in relation to IPV occurrence (Sutton et al., 2021). It is important to reinforce that ending the relationship is not always an individual’s goal; SGM may seek services to reduce their role in bidirectional IPV and learn healthier coping mechanisms to shift and maintain the dynamic of their intimate relationship(s). Such services are also intrinsically linked with supporting individuals experiencing substance use challenges, providing clients with skills and resources to reduce or eliminate such behaviors.

### Gaps in Understanding Mental Health Service Access for SGM Who Experience IPV

Mental health providers can use various strategies to support individuals experiencing IPV. Cognitive behavioral therapy (CBT) focuses on shifting one’s way of thinking and behaving by enhancing thought recognition, behavioral understanding, problem-solving, and self-efficacy. In a meta-analysis of 12 randomized controlled trials, Tirado-Muñoz et al. (2014) found that CBT was effective in reducing physical and psychological, but not sexual or unspecified, IPV occurrence among women. Similarly, advocacy-related approaches such as empowerment sessions and social support were found to reduce physical and psychological, but not sexual or any, IPV occurrence among women (Tirado-Muñoz et al., 2014). Eye movement desensitization and reprocessing techniques, a strategy that simultaneously engages clients to reflect on traumatic experiences and stimulates them via auditory or visual cues, has been shown to significantly decrease posttraumatic stress disorder symptoms (Vereecken & Corso, 2024), including those who experience sexual IPV (Rothbaum, 1997). Findings regarding interventions focused on people who cause harm, including CBT and psychoeducation, are contradictory, while those targeting couples as opposed to individual partners show no difference (Babcock et al., 2024; Stover et al., 2009). Although mental health services may support individuals experiencing IPV, findings are equivocal, especially for groups diverging from the cisheterosexual identity, such as SGM.

Indeed, research suggests that mental health providers are often not trained to provide optimal responses to SGM who experience IPV (Shali, 2024). Licensed clinicians often report being inadequately trained to support SGM with IPV-related concerns (Alston et al., 2021), stemming from training and education founded on cisheterosexual dynamics that lead to multiple myths and misconceptions when translated to SGM relationships (Kurbatfinski et al., 2024b). Although strategies such as CBT exist to effectively support cisheterosexual individuals experiencing IPV, the effects are undermined when applied among SGM due to stigma, cultural incompetence, and an inability for providers to adequately integrate sexual and gender diversity into service delivery (Shali, 2024). Consequently, mental health providers may exacerbate challenges in SGM IPV healing journeys when not attuned to the unique needs and experiences of SGM (Sutton et al., 2021). Therefore, to be effective when working with this population, mental health providers need to be trained to provide services to SGM while considering unique manifestations of IPV related to sexual and gender diversity and compounding forces such as homophobia (Kimmes et al., 2019). In fact, when affirmative sexual and gender diversity language is integrated in training and service delivery, strategies such as CBT become powerful interventions to support SGM experiencing IPV (S. A. Carvalho et al., 2022). Identifying characteristics of mental health services that promote or reduce the quality of care for SGM experiencing IPV is paramount to reducing harm and promoting health equity.

### Purpose of the Study

In a recent review, mental health services were identified as the most helpful type of formal support for IPV help-seeking among SGM relative to legal supports, police, and medical professionals (Kurbatfinski et al., 2023). Although mental health service providers are often viewed as helpful sources of support for SGM experiencing IPV, the findings are not straightforward (Kurbatfinski et al., 2023). This systematic review is the first study to synthesize existing literature on SGM experiences with mental health supports related to IPV in North America. More specifically, authors aimed to answer the following research question: “What characteristics of mental health services promote or worsen the experiences of SGM accessing supports for IPV in North America?” Identifying clear strengths and limitations will provide opportunities to improve the quality of care provided by formal mental health services for SGM who experience IPV while also informing organizational and governmental policymakers.

## Methods

This systematic review used secondary data from a larger study focused on examining the help-seeking experiences of SGM exposed to IPV (Kurbatfinski et al., 2023). The research team developed the review in accordance with the Joanna-Briggs Institute (JBI) guidelines (Peters et al., 2015) and reported it according to Preferred Reporting Items for Systematic Reviews and Meta-Analyses (PRISMA) (Moher et al., 2009).

### Terminology

In this review, the term IPV refers to all physical and non-physical forms of violence or abuse of varying levels of severity and frequency. Furthermore, in lieu of terms such as “victim” or “survivor”, which have been described as stigmatizing and disempowering by some individuals with lived experience, authors used adapted versions of “individuals who experience IPV.” Similarly, instead of the term “perpetrator,” authors used “those who cause harm or use abusive behaviours” when engaging in IPV. These terms are also reflective of the often, bidirectional nature of IPV, as many individuals may both experience and use IPV (Herbert et al., 2020; Hine et al., 2022; Shields et al., 2025). In addition, the term SGM encompasses the wide range of sexual orientation and gender identity minority groups, serving as an inclusive term for this review.

### Identifying Relevant Studies

An academic librarian with experience in searching to support knowledge syntheses (MM) conducted a structured search to identify candidate articles. This author constructed a strategy for MEDLINE (OVID) using Medical Subject Headings (MeSH), keywords, and Boolean operators to combine the concepts of 2SLGBTQQIA+ people and intimate partner violence/abuse. Examples of the search terms used to identify SGM included MeSH terms such as Homosexuality, Bisexuality, Transsexualism, and Health Services for Transgender Persons, as well as keywords such as “gay,” “pansexual*,” “Two-Spirit*,” “lesbian*,” “genderqueer,” and “men who have sex with men.” IPV-related search strings included the MeSH terms Intimate Partner Violence, Sex Offenses, Rape, Violence, and Physical Abuse in addition to an extensive list of keywords such as “rape” or “violence” or “abuse” adjacent to “spousal” or “partner*” or “dating.” We also made sure to identify inclusive terms to refer to intimate partners or intimate relationships, including the terms “casual” or “informal” or “anonymous” adjacent to “sex” while also searching for expressions such as “friend? with benefits” and “hookup?” A sample search strategy with a full list of terms used is provided (Supplemental Material 1). The original MEDLINE strategy was translated to Embase (OVID), PsycInfo (OVID), Scopus (Elsevier), CINAHL (EbscoHost), Genderwatch, and Social Science Abstracts, completing searches in the databases from inception to June 27, 2024.

### Inclusion and Exclusion Criteria

To be included in this review, studies had to be published in peer-reviewed journals, be conducted in North America (specifically the United States or Canada), be published in English or French (reflecting the linguistic capacity of the team), be observational in nature (utilize studies that collected quantitative, qualitative, or mixed methods data from participants), and describe at least one help-seeking experience of a SGM individual who experienced IPV and contacted a mental health provider, or the experience of a mental health provider with lived experience of IPV who also provided support to those experiencing IPV. Experiences with mental health providers included any occurrence in which an individual experiencing IPV attempted to access or accessed a psychologist, psychiatrist, counselor (couples or individual), therapist, social worker, or peer support group (with a professional facilitator). This review excluded dissertations (due to limited resources), conference abstracts, commentaries, opinion letters, editorials, gray literature, and intervention studies. Studies focused solely on barriers to accessing mental health services or disclosure prevalence were also excluded, as these have been reviewed elsewhere (A. F. Carvalho et al., 2011).

### Study Selection Process

**Figure 1.**
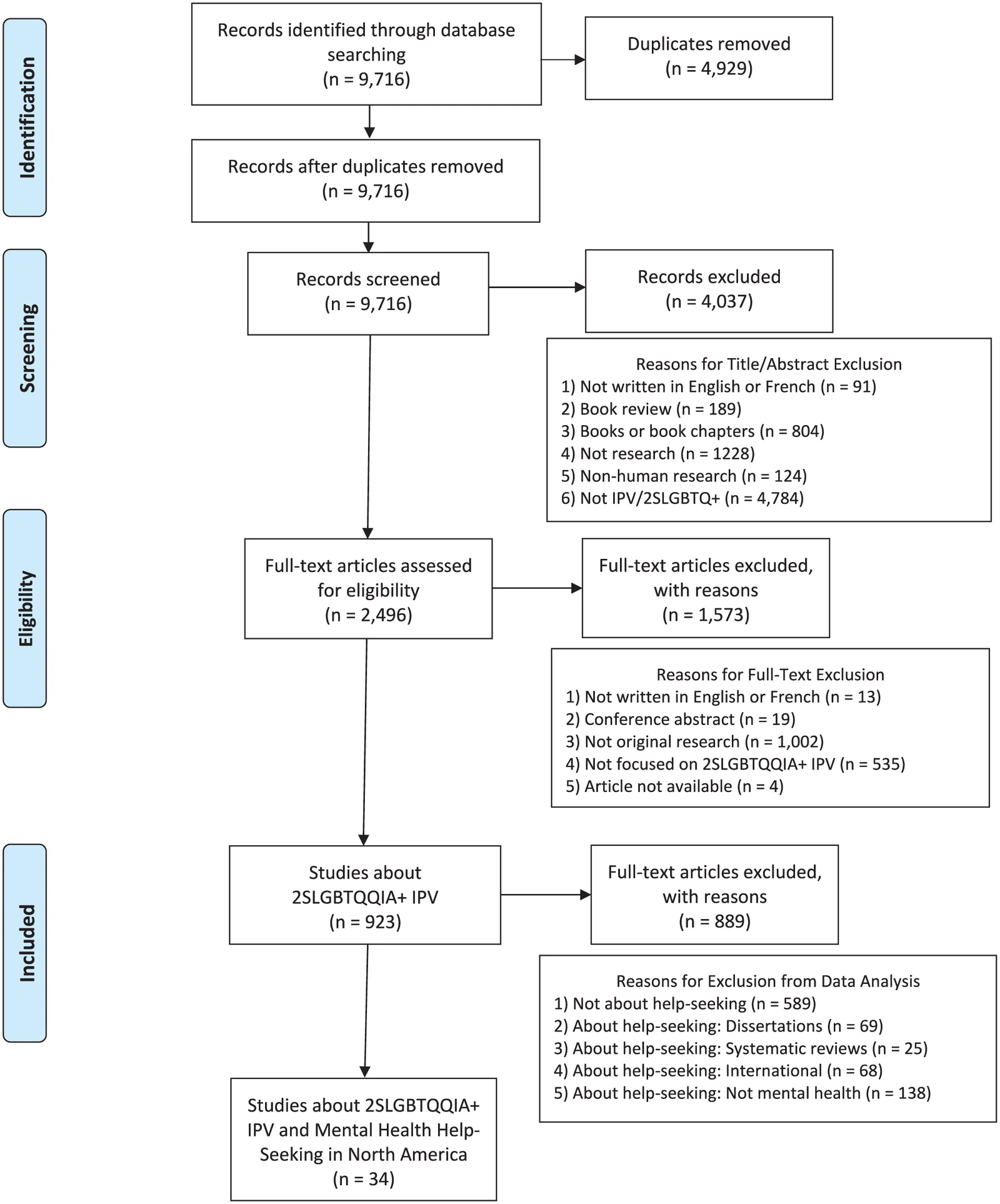
PRISMA flow diagram. *Note*. PRISMA = Preferred Reporting Items for Systematic Reviews and Meta-Analyses.

JBI meta-aggregation methods guided data collection alongside standard systematic review methods (Lockwood et al., 2015). The authors used EPPI-Reviewer to store, organize, and screen the evidence sources (Thomas et al., 2023). We screened 9,716 references on title and abstract, 2,496 references on full text, and located 923 studies focused on SGM IPV. Of these, 34 studies met the inclusion criteria for this review and described at least one experience of seeking help from a mental health practitioner. Authors provide a PRISMA flow diagram outlining the article selection process along with reasons for full-text exclusions (Figure 1).

### Data Extraction and Qualitative Synthesis

This review used an adapted data form from the larger study (piloted and completed in Excel) to extract the following data from the included studies: (a) study information (e.g., methods, study design, location of study, publication date); (b) study objective(s); (c) participant demographic information; (d) service provider information; (e) types of IPV experienced; (f) prevalence and outcomes of accessing mental health services; (g) barriers and facilitators to accessing mental health supports; and (h) recommendations. Two authors (NU, SK) independently reviewed and extracted data from the included studies onto the Excel form.

JBI meta-aggregation methods also guided data analysis (Lockwood et al., 2015). These methods provide a thorough approach to synthesizing qualitative evidence from studies, such that singular findings/themes are first identified, then grouped into categories, which are then further grouped into key synthesized findings. Using an approach resembling thematic analysis, one author (SK) identified findings (themes) from the included studies and organized them in an Excel file. Following this, the same author combined the themes to create categories based on similarities and commonalities (e.g., outcomes of accessing mental health services, positives observed in mental health service provision) and subsequently combined these categories to develop a few synthesized findings. Three other authors (KM, NU, ZM) reviewed the analyzed data for accuracy and clarity based on the extracted data.

### Critical Appraisal Methods

Two authors (NU, SK; 90% inter-rater agreement) used the JBI Critical Appraisal Checklists to assess the rigor and quality of the included studies, providing some inference on their degree of bias (Joanna Briggs Institute, 2017). The qualitative (10 questions) and cross-sectional (8 questions) checklists permitted appraisal of qualitative and quantitative studies, respectively; and mixed-method studies were assessed using both checklists. The following options were possible responses for each question: “yes”; “no”; “unclear”; and “not applicable.” The authors used the critical appraisals to provide an assessment of the quality of the included studies rather than to exclude them, resolving any disagreements via consensus.

## Results

Of the 34 extracted and analyzed studies, 29 were conducted in the United States and 5 were conducted in Canada. Most were qualitative (*n* = 21), followed by quantitative (*n* = 9) and mixed-method (*n* = 4) studies. Fifteen studies were completed between 2020 and 2024, six between 2015 and 2019, five between 2010 and 2014, three between 2005 and 2009, and the remaining five during or before 2000. Most qualitative and mixed-method studies (15/25) described their qualitative methodologies, including intersectionality (*n* = 3; Lippy et al., 2020; Simpson & Helfrich, 2014; Soares et al., 2024), narrative (*n* = 3; Freeland et al., 2018; Hereth, 2021; Kurdyla, 2023), feminist (*n* = 2; Anderson & Overby, 2020; Wang, 2011), queer and feminist (*n* = 1; de Heer et al., 2024), participatory action research (*n* = 1; Bornstein et al., 2006), help-seeking (*n* = 1; Hardesty et al., 2011), ecological (*n* = 1; Roy et al., 2024), exploratory (*n* = 1; Oueis et al., 2024), trans methodology (*n* = 1; Shultz, 2019), and minority stress (*n* = 1; Jackson et al., 2017) frameworks. Nine studies specifically examined help-seeking in relation to sexual IPV (Anderson & Overby, 2020; Coston, 2019; de Heer et al., 2024; Donne et al., 2018; Jackson et al., 2017; Oueis et al., 2024; Todahl et al., 2009; Walsh et al., 2025; Wang, 2011).

Sample sizes of included studies ranged from 1 (case study; Shultz, 2019) to 2,462 (national survey; Lippy et al., 2020) and age, when provided, ranged from 15 (average; Wright et al., 2023) to 50 to 60 (range; Wang, 2011) years. Only nine studies focused primarily (90% or more) on one sexual orientation and/or gender identity group that was well-defined. Some studies (*n* = 17) included primarily white participants, while others (*n* = 10) focused on racially mixed samples, and fewer (*n* = 3) included a predominantly racialized sample; four studies provided no details related to race/ethnicity. When reported, average, median, or median income levels in all study samples (*n* = 11) were typically around $50,000 or less (either USD or CAD). Conversely, when reported, educational attainment was relatively high in all studies (*n* = 17; at minimum participants attended some college, university, or trades education). See Supplemental Material 2 for additional information on each study.

**Figure 2.**
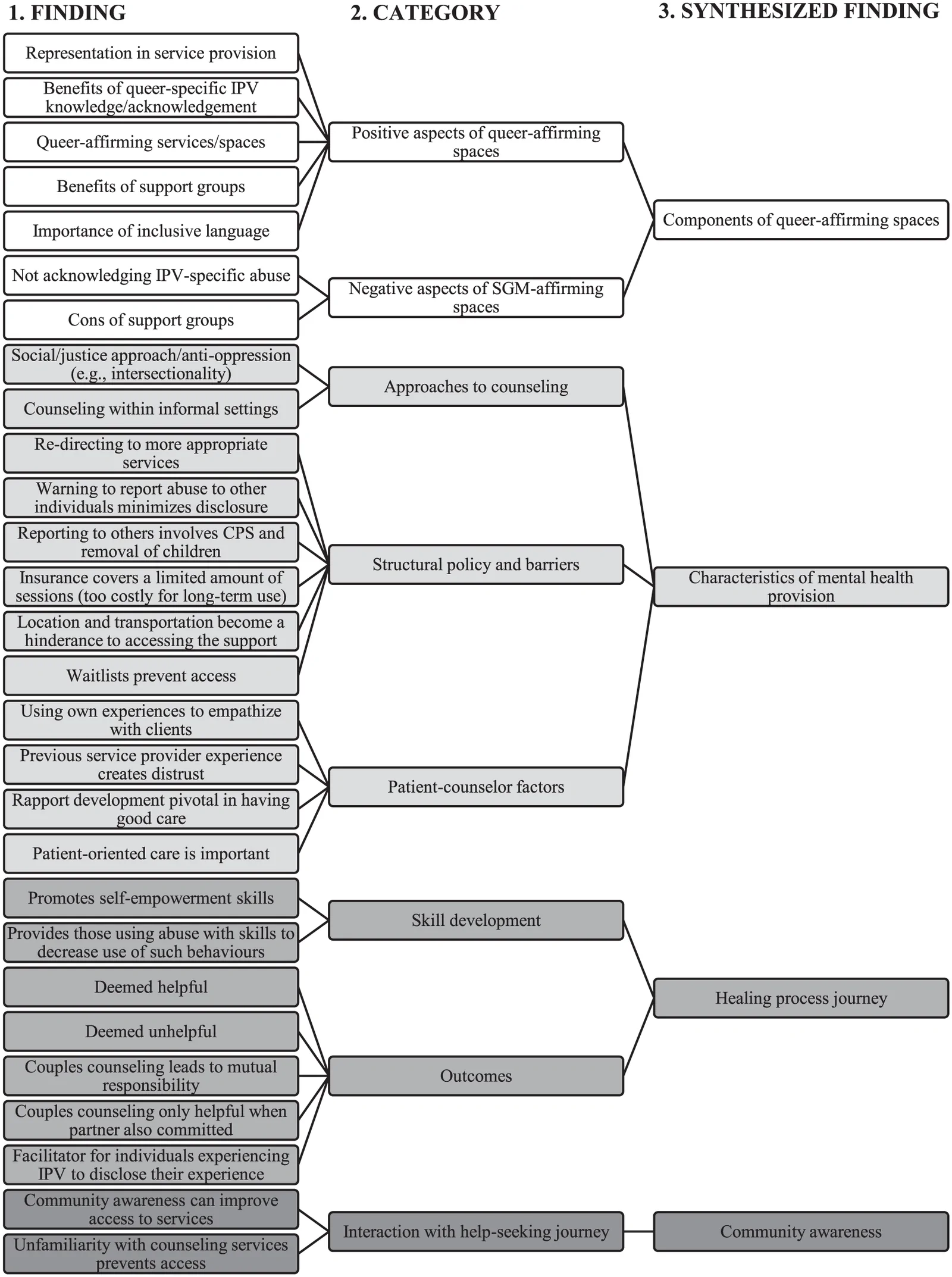
Visual depiction of meta-aggregation. The authors first identified 28 findings, which were organized into eight categories, producing four main synthesized findings.

Through meta-aggregative methods, authors identified 28 unique findings, which were organized under eight categories. These eight categories were ultimately combined to result in four key synthesized findings (Figure 2), reflecting concepts derived from the results, including (a) components of SGM-affirming spaces, (b) characteristics of mental health provision, (c) healing journey process, and (d) community awareness and familiarity with services. Critical findings are reported in Table 1.

**Table 1.** Critical Findings.

• SGM who experience IPV most often describe mental health supports as helpful, especially relative to other formal sources of support (e.g., police, doctors, domestic violence shelters) • SGM-affirming mental health spaces and SGM-IPV specific training, knowledge, and acknowledgment among professionals underlie acceptability and comfort among SGM seeking help related to IPV experiences; an intersectional approach can assist providers to better understand how other experiences related to, and beyond, sexual and gender identity compound experiences of IPV • Mandatory reporting laws hinder the disclosure of IPV among SGM individuals • Mental health support groups were described as both positive and negative; they can promote disclosure through shared experience and relatability, but also trigger re-traumatization and discomfort • Couples counseling was not identified as supportive unless the partner is also committed to the help-seeking journey • Community awareness of SGM-specific IPV resources and services is an important factor that contributes to the acceptability and accessibility of mental health supports

*Note*. IPV = intimate partner violence and abuse; SGM = sexual and gender minorities.

### Components of SGM-Affirming Spaces

SGM-affirming spaces are environments which promote representation of SGM identities among mental health service providers, provide resources specific to the needs of SGM, train and specialize staff in the unique manifestation of IPV among SGM alongside other traumas, and utilize inclusive language. Positive and negative aspects of SGM-affirming spaces were highlighted in the findings. In fact, SGM who experienced IPV “noted that they would not have sought support from a program that was not queer-specific” (Bornstein et al., 2006, p. 172). Having mental health professionals who were knowledgeable about and could detect IPV more generally was at the very least necessary (Brandt & Stults, 2024; Drouillard & Foster, 2024), but most conducive to effective counseling environments were professionals with SGM-specific IPV knowledge who acknowledge the violence and abuse (*n* = 8; Anderson & Overby, 2020; Bornstein et al., 2006; Coston, 2019; de Heer et al., 2024; Oswald et al., 2010; Renzetti, 1988; Shultz, 2019; Walsh et al., 2025; Wang, 2011). One study revealed that “the two most important factors [were] . . . whether the mental health professional addressed the dynamics of their same-sex relationship and their experiences with IPV” (Oswald et al., 2010, p. 293). Furthermore, providing relevant resources or connecting SGM individuals with other mental health supports (e.g., therapists) was also viewed as beneficial (Whitton et al., 2024). Conversely, several studies (*n* = 6) emphasized the negative effects of mental health providers not acknowledging SGM-specific IPV (Bornstein et al., 2006; Freeland et al., 2018; Jackson et al., 2017; Oswald et al., 2010; Renzetti, 1988; Simpson & Helfrich, 2014), explaining how this led to minimization of SGM participants’ experiences and ultimately hindered help-seeking behaviors.

The sexual and gender identity of mental health service providers was seen as an important factor for many individuals (Freeland et al., 2018; Walsh et al., 2025). Having SGM representation among service providers fostered a level of comfort and security: “some participants believed that a good gay friendly counselor [participant ID] was an excellent choice for counseling services because it helped them get through difficulties in their life” (Freeland et al., 2018, p. 305). The presence of SGM mental health service providers is highly interconnected with training and awareness regarding how different SGM use and experience IPV. In relation to this, inclusive language was emphasized as an important factor (Walsh et al., 2025), whereby “heterosexist language [was] a significant barrier” that prevented individuals from disclosing “the truth” (Simpson & Helfrich, 2014, p. 459).

Support groups were included in this synthesized finding as they consist of similar identifying SGM and thus can be SGM-affirming spaces. For example, trans identifying people may feel encouraged to disclose their experiences in trans “support groups [because] in this environment, one can assume their gender identity is supported” (Kurdyla, 2023, p. 484); this shifts the focus from disclosing one’s identity to being able to talk about IPV. However, for some, support groups may be counterproductive. This was emphasized in relation to sexual violence, where a participant described how “he found it very traumatic at the beginning because people would go into very specific details, more than he and other people could handle” (Donne et al., 2018, p. 197). Overall, mental health spaces were generally perceived as beneficial for SGM experiencing IPV (though less so when obtained through couples counseling) and described as more effective when in an SGM-affirming space.

### Characteristics of Mental Health Provision

According to the studies in this review, mental health service provision is affected largely by the professional approach and rapport established by the practitioner, alongside structural policies and barriers. Two approaches were identified in relation to mental health service provision: intersectionality (anti-oppressive framework) and counseling in an informal setting. Intersectionality recognizes the importance of how oppressions related to sexuality, gender, race, and disability intersect and influence experiences of IPV (Crenshaw, 2017); failing to address interlocking oppressions can result in suboptimal care, that is, “having to prioritize . . . personal problems in order to receive services negatively impacted . . . health . . .” such that having “more comprehensive services” (Simpson & Helfrich, 2014, pp. 455–456) was needed to tackle the intersecting oppressions of income and race, alongside others, with mental health illness. Furthermore, racialized people who also identify as SGM emphasized “finding someone . . . who’s culturally competent to the identities that you have” so that they do not have “to explain the social or cultural aspects of sex and sexual violence to a therapist and especially someone of an opposite gender” (Walsh et al., 2025, p. 3). Since counseling services focus largely on providing mental health supports, redirection to other services may be required if professionals cannot address the needs of their clients; this occurred in one study and was seen as a positive experience when accessing mental health services (Hardesty et al., 2011). However, mental health service provision within an informal setting may relieve the pressure and expectations associated with professional mental health environments: “while most counseling services are perceived to be in a formal counseling center/office with mental health professionals, many participants also suggested counseling in informal settings, such as churches” (Freeland et al., 2018, p. 305), reflecting another avenue to accessing mental health support.

Structural mental health service provision policies can serve to facilitate or hinder the help-seeking journeys of SGM experiencing IPV. Multiple accessibility challenges were identified as barriers to accessing mental health services, including location and transportation (e.g., “not being able to drive and living far away from where supportive services are offered” Anderson & Overby, 2020, p. 1571), price and insurance coverage (e.g., actual costs or insufficient insurance coverage; Whitton et al., 2024), and waitlists (Anderson & Overby, 2020). In relation to infrastructure sufficiency, participants “touched on how it was difficult to find a psychotherapist due to a lack of community resources specific to” SGM and “mental health services in general” (Oueis et al., 2024, p. 420); this was amplified for SGM requiring mental health supports in violence shelters (Soares et al., 2024). Long waitlists and insufficient infrastructure also impeded mental health journeys for university students (Walsh et al., 2025). Mandatory reporting laws (laws that require mental health professionals to report a client’s experiences if placing themselves, others, or the community in danger) served as another barrier or exacerbator of IPV impacts. Though these laws are intended to establish safety for the person disclosing violence or abuse, and for those in close contact (e.g., children), they can also lead SGM experiencing IPV to “withhold information and/or misrepresent their experiences” and prevent receiving “help based on the entirety of their experience” (Lippy et al., 2020, pp. 261–262). Individuals also explained how pursuance of these laws led to significant consequences including “involvement with the criminal legal system and removal of their children” (Lippy et al., 2020, pp. 262–263); in fact, the fears pertaining to possible outcomes from mandatory reporting laws prevented help-seeking among nearly 25% of individuals (Lippy et al., 2020), revealing decreased opportunity for SGM to receive IPV-specific mental health support.

Developing rapport with a mental health provider was also instrumental for some individuals in discussing their experiences (de Heer et al., 2024; Donne et al., 2018). This was particularly relevant in situations where SGM had pre-established rapport with their mental health provider for other stressors they experienced (e.g., school stress; Donne et al., 2018). Once rapport was established, “they often disclosed and talked openly about sexual violence experiences as well” (Donne et al., 2018, p. 197). Some SGM met more than one counseling professional before finding the right fit (de Heer et al., 2024). Previous negative encounters with counseling supports also led to mistrust of engaging counselors in the future, and delays accessing such supports (Jackson et al., 2017). Some service providers used their own IPV experiences to align with SGM accessing mental health support: “their unique role as a service provider and survivor did influence their sensitivity for asking survivors they worked with whether these were barriers in seeking support” (Anderson & Overby, 2020, p. 1570). Mental health support was also described as “victim oriented,” offering an opportunity for “therapy with mental health professionals, volunteer counseling services at churches, and individual and group counseling services” (Freeland et al., 2018, p. 305) to cater to the unique experiences of diverse SGM accessing such supports; however, the lack of consideration of potential IPV bidirectionality in causing and experiencing harm as a barrier to access was not explored. Some individuals identified the consequences of therapists using misguided, non-patient-oriented approaches: “I think in the first of those two sessions it was about finding the good that the relationship had taught me about myself, which I just wasn’t ready for. So, I just stopped going” (Brandt & Stults, 2024, p. 183). Ultimately, many factors contributed to experiences with mental health providers, reflecting the nuances related to effective counseling provision.

### Healing Journey Process

In addition to other outcomes, personal development was described in relation to the healing journey process. When discussing general impressions of mental health services, these were (a) identified as facilitators for disclosure of IPV experiences (Brandt & Stults, 2024; Kurdyla, 2023) and (b) predominantly portrayed as helpful (*n* = 12; Brandt & Stults, 2024; Coston, 2020; de Heer et al., 2024; Jackson et al., 2017; Kurdyla et al., 2021; Merrill & Wolfe, 2000; Oueis et al., 2024; Renzetti, 1988; Schilit et al., 1991; Turell, 1999; Turell & Cornell-Swanson, 2005; Whitton et al., 2024; Wright et al., 2023) more than unhelpful (*n* = 3; Brandt & Stults, 2024; Donne et al., 2018; Scherzer, 1998). In one study, there was an indication that SGM may first engage “in maladaptive coping strategies (e.g., self-harm, unhealthy substance use) before beginning therapy” (Oueis et al., 2024, p. 420). One of the studies that suggested that mental health services were unhelpful mainly contextualized this finding in relation to couples counseling, whereby individuals identified feelings of “guilt” when their partner was “attacked” or “being threatened” (Scherzer, 1998, p. 41). Reduction of IPV and promotion of healthy relationships was described as not feasible when “the partner would not participate in some way to work together through the issues” (Scherzer, 1998, pp. 40–41); however, when both partners worked together, the following outcomes were identified: “they learned better communication skills, ‘things worked out’, they learned to set better boundaries” (Scherzer, 1998, pp. 40–41). Lastly, some mental health providers suggested leaving the abusive partner (Whitton et al., 2024); such advice may or may not be beneficial based on what the SGM individual is seeking.

Accessing mental health supports also fostered self-development (Brandt & Stults, 2024; Freeland et al., 2018; Jackson et al., 2017; Oueis et al., 2024; Roy et al., 2024; Scherzer, 1998). More generally, mental health supports helped SGM experiencing IPV to acknowledge their experiences and to actively address the problem (Roy et al., 2024). In one study, “participants stated that counseling could be useful to assist with self-improvement and increase self-love and self-care” (Freeland et al., 2018, p. 305), enabling individuals experiencing IPV to overcome internal and external criticism. Some individuals were able to realign with their interests and return to activities that they previously enjoyed before experiencing IPV (Brandt & Stults, 2024). Others were able to develop the capacity to more effectively cope with their emotions rather than engaging in avoidance (Oueis et al., 2024). Another considered self-development from the perspective of those causing harm, where participants contextualized their abusive behaviors in relation to childhood experiences (e.g., experiencing incest, witnessing parental substance use) that disadvantaged them (Scherzer, 1998). Mental health services (alongside other formal sources of support) provided these individuals with skills that “helped create boundaries and tools for coping [and] facilitated better communication” (Scherzer, 1998, p. 41). Nevertheless, it is important to state that even those who access mental health supports for an extended amount of time (e.g., 5 years) may still experience longer-term impacts (Oueis et al., 2024).

### Community Awareness and Familiarity With Services

Increasing community awareness was underlined as a necessary facilitator for SGM experiencing IPV to obtain needed supports (Freeland et al., 2018; Kurdyla, 2023; Ollen et al., 2017; Simpson & Helfrich, 2014; Todahl et al., 2009). A reported barrier to accessing mental health services was a “lack of confidence in the mental health resources available” (Ollen et al., 2017, p. 116). Similarly, another study reported that a lack of “familiarity or comfort with mental health services” (Hereth, 2021, p. 470) prevented or restricted access. To address this problem, “IPV community education and outreach efforts ‘were recommended’ to better raise awareness about IPV and expose survivors to possible resources” (Kurdyla, 2023, p. 489). Though speaking more broadly about IPV and social service organizations, one study further noted SGM participants lacked awareness of IPV-specialized supports “despite the fact that their city houses one of the most comprehensive LGBTQ social service agencies in the country” (Simpson & Helfrich, 2014, p. 456). More relevant to university settings, students portrayed the ways mental health providers failed to share university resources specific to individuals experiencing IPV (Walsh et al., 2025). Therefore, a need for increased awareness of IPV-related services was identified across many service provision settings.

### Critical Appraisal Results

Most studies were rated moderate to high in quality (Supplemental Material 3). The item least often answered “yes” among qualitative studies was “is the influence of the researcher on the research, and vice-versa, addressed?” The items least often answered “yes” among cross-sectional studies were “was the exposure measured in a valid and reliable way?,” “were confounding factors identified?,” and “were strategies to deal with confounding factors stated?”

## Discussion

Using meta-aggregative methods, this appears to be the first systematic review to synthesize existing North American literature on the help-seeking experiences of SGM accessing mental health services related to IPV. It focused on experiences with mental health service providers rather than anticipated barriers to accessing such services. Four main synthesized findings were identified, including components of SGM-affirming spaces, characteristics of mental health provision, the healing journey process, and community awareness and familiarity with services. SGM-affirming spaces consisted of mental health service provider environments that promoted SGM representation, use of inclusive language, and SGM-specific IPV resources; underlying all of this, awareness and acknowledgment of SGM-specific IPV were instrumental in increasing comfort and acceptability of mental health service utilization. In terms of characteristics of mental health provision, approaches to service provision and the importance of rapport with the service provider, along with structural policies and barriers, were described. The healing journey was contextualized in relation to overall helpfulness and self-development, with less optimal findings in the context of couples counseling. Lastly, community awareness of IPV was identified as a pivotal factor that facilitated help-seeking and overall experiences with accessing mental health services.

A systematic review conducted by Sutton et al. (2021) on IPV training for mental health service providers identified some overlapping findings, such as the importance of training, collaboration between agencies, sensitive responses to disclosures, and patient-client rapport; however, this current review significantly extends their findings by focusing on help-seeking experiences of those experiencing IPV and by increasing generalizability to SGM, a community underserved by current mental healthcare services.

### Characteristics of Inclusive and SGM-Affirming Mental Health Services

Broadly stated, SGM-affirming mental health services attend to inclusion, sensitivity, and awareness of SGM-specific IPV and societal challenges. Access to mental health service providers with similar identities is imperative to promote comfort when SGM accessing support. Mental health providers working alongside SGM experiencing IPV explained how trauma stemming from discrimination and oppression compounded SGM help-seeking journeys (Alston et al., 2021). Mental health providers who have experienced similar forms of marginalization (e.g., transmisogyny) are more likely to have an increased understanding of how these experiences interact with SGM behaviors and help-seeking journeys, enabling more appropriate service provision. Mental health professionals have ethical obligations to seek training to recognize and acknowledge SGM-specific forms of IPV, such as gender invalidation, outing, and deadnaming, to ensure that those accessing mental health supports feel heard and validated (Bermea et al., 2019; Furman et al., 2017). Failure to do so results in minimization of SGM experiences, which may generate feelings of confusion, uncertainty, and shame (Overstreet & Quinn, 2013), alongside a rupture in the relationship with the service provider, including termination.

From an intersectional lens (Crenshaw, 2017), SGM who are also racialized, experiencing ableism, or living in disadvantageous socioeconomic conditions must navigate additional oppressive social forces alongside their SGM identity (Kattari et al., 2015, 2017; Kimmes et al., 2019); mental health providers who align with these identities or have navigated similar experiences are instrumental in promoting inclusive and SGM-affirming mental health services, especially when combined with SGM-specific IPV training and awareness, SGM service provider representation, and SGM-specific resources.

### Structural Barriers and Counselor Attributes

Mental health service provision is affected largely by structural policies and barriers, in addition to the professional approach and rapport established. Sherman et al. (2022) reported that Black trans women experienced violence from multiple individuals (i.e., poly-victimization; Finkelhor et al., 2007), as a function of three barriers: cost, accessibility, and rapport with their healthcare professional. SGM continue to experience mental health inequities via discriminatory social forces, by which participation in the labor market and income are reduced, effectively hindering access mental health supports (Waite et al., 2020); paradoxically, without adequate mental health support, these individuals are less likely to receive the care they need to contribute to the labor market. This negative feedback loop is further exacerbated when considered in the context of intersectionality. One study in this review emphasized that service providers who themselves previously experienced IPV were more cognizant of these barriers and willing to openly discuss them with their clients (Anderson & Overby, 2020), reflecting a means of reducing the impact of structural barriers on SGM mental health support. Such relational strategies promote sensitivity, respect, and patient-oriented care while also engaging strategies to lessen the burden of these structural barriers. By contrast, laws such as mandatory reporting, which require both reporting an incident of IPV to the criminal justice system and identifying which member of the couple caused the harm and which partner experienced it, are meant to increase access to support when IPV occurs. However, the impact of mandatory reporting has been called into question for identity-based assumptions and structural reproduction of prejudice and inequity, as well as their origins in retributive justice (Dosanjh et al., 2008); the potential negative effects likely intersect with SGM identities due to discriminatory social forces and biases.

Thus, integrating an intersectional and anti-oppressive lens is essential to improving mental health supports for SGM who experience IPV (Bowleg, 2012). Originating in Black feminism, intersectionality theory encapsulates how one’s identities simultaneously interact with the surrounding societal factors and social forces to either advantageously or disadvantageously impact individuals’ and communities’ life experiences (Crenshaw, 2017). Disentangling experiences of intersecting oppressions within the context of SGM IPV can enable mental health providers and their organizations to reflect on and situate clients and providers more equitably in their professional relationships. Service providers’ use of their own IPV experiences within the therapeutic context can be another means of addressing such relational boundaries through empathy; however, ethical practice by mental health professionals necessitates exercising caution when doing so (especially when not of similar identity groups) as to not take away from the client’s experience or story (Aponte, 2022). These ideas are foundational for the development of strong rapport between mental health professionals and clients (Flückiger et al., 2018), which is commonly described as one of the most important factors in optimizing mental health provision.

### How Can Mental Health Service Provision Affect the Healing Journey?

With one exception, findings from this review reinforced the benefits of mental health services for SGM experiencing IPV. It is expected that individuals accessing such services will only pursue them when feeling a safe and reliable connection with their mental health provider; for SGM, challenges accessing mental health supports may be exacerbated due to the increased likelihood of misunderstanding, stigma, and discrimination experienced previously in healthcare settings (Kurbatfinski et al., 2024b). Therefore, the positive outcomes of mental health services in the context of SGM IPV can likely be attributed to receiving client-centered care (Sorrentino et al., 2021), particularly when the provider and service user share similar identities. Furthermore, self-development was identified as a contributor to healing in this review. Social forces such as homophobia are exclusive to the SGM community, are internal and external to the community, and perniciously prevent self-growth (Meyer, 2003). The effects can result in poorer mental health (e.g., depression, anxiety), a risk factor for entering abusive relationships (Herbert et al., 2020); perception of self and mental health status can further deteriorate if vulnerabilities become the context for psychological and emotional abuse (Herbert et al., 2020). The interconnection between mental health, self-perception, and IPV represents a negative feedback loop that requires substantial effort to reverse. Nevertheless, mental health interventions such as CBT may be a favorable approach to promoting self-healing following IPV and can be applied to address the unique experiences of SGM (Pachankis, 2018). Experiences with couples counseling were described as mainly negative, highlighting feelings of guilt or unequal commitment to repairing the relationship (Scherzer, 1998). It should be noted that the study by Scherzer is outdated and reflective of only two participants, pointing to the need for further research on this topic.

### Increasing Community Education and Awareness of IPV is Imperative for SGM

For SGM, community awareness was an important discourse that interconnected with individuals’ experiences of using mental health services following IPV. Public campaigns to raise awareness of IPV rarely contain information about SGM-specific forms of harm, data on SGM IPV prevalence and incidence, or IPV-related mental health issues in general or specific to members of SGM communities. Furthermore, links to SGM services and training for service providers are difficult to find within public IPV information and resources, which prioritize violence against cisgender heterosexual women (e.g., Government of Canada, 2023). Available resources, particularly on the internet, are often geographically limited and urban-centric (e.g., Quinn, 2019). Education is needed to acknowledge specific forms of SGM IPV by individuals experiencing IPV and by mental health service providers. Furthermore, without awareness of, or access to, relevant services, SGM who actively seek mental health supports may be discouraged from future follow-up. Therefore, greater community IPV education and resource awareness are needed within SGM communities and mental health service provider communities.

### Implications for Practice and Policy

Based on the findings of this review, the authors provide several implications for practice and policy. Given the largely positive experiences of SGM with mental health supports in the context of IPV, governments and policymakers should prioritize resource allocation to expand and diversify available services and reduce cost, waitlists, and transportation barriers to promote accessibility. In addition, mental health providers and organizations should be required to undertake multiple mandatory training modules to ensure providers are attuned and sensitive to SGM seeking help for IPV and to further ameliorate service provision. To increase representation and SGM-affirming mental health supports, policies are needed to increase educational opportunities and scholarships for SGM to obtain relevant experience to become a mental health professional, increasing SGM provider representation and accessibility for SGM experiencing IPV. These recommendations extend to members of other marginalized communities (e.g., disability) to ensure that full representation is available within the mental health sector.

Changes to mandatory reporting laws should also be explored due to (a) barriers to SGM and non-SGM IPV help-seeking experiences and (b) the disproportionate impacts mandatory reporting laws incur on marginalized groups. Mental health support groups should be facilitated by professionals with several other resources available to limit re-traumatization and to promote the navigation of IPV experiences. In addition, bi-directional IPV is often misunderstood within the SGM narrative; couples counseling must be reshaped to ensure that bi-directional IPV is considered without blame, minimization, or stigmatization. Lastly, policies should be put into place that increase advertisements and outreach within different settings (e.g., high school) to ultimately increase awareness of available mental health supports for SGM experiencing IPV.

### Implications for Future Research

Researchers in the field of SGM IPV and mental health should evaluate and adapt existing IPV interventions (e.g., CBT) for SGM who seek mental health supports related to IPV. More research is needed to illuminate how SGM experiences with mental health supports vary based on each unique identity group; most studies are comprised of small, stratified groups, resulting in inadequate sample sizes to draw appropriate conclusions. Furthermore, this study focused on North American literature as preliminary examination of included studies appeared to be concentrated in this region. Authors suggest that researchers in other regions conduct research on SGM experiences accessing mental health supports, as cultural differences may further influence how formal service providers providing support to SGM.

### Limitations

As the SGM community includes a diversity of sexual and gender identities, the findings should be carefully considered to ensure applicability; however, findings from this study encapsulate themes that can provide initial insights into the experiences of each respective group. Authors of this review combined findings from different service mental health provider types; though some findings may not necessarily be generalizable to each provider type, this was deemed the best approach due to limited existing literature. Most studies included in this review were conducted in the United States, decreasing the generalizability of findings to other geographic regions. Therefore, findings will be most generalizable to settings with similar demographic, funding, and policy environments.

## Conclusion

To our knowledge, this is the first systematic review to use meta-aggregative methods to synthesize literature on the help-seeking experiences of SGM accessing mental health supports related to IPV in North America. Mental health supports were generally viewed as positive by SGM and as an important resource when experiencing IPV. Experiences of SGM accessing mental health services were categorized under four key themes: (1) components of SGM-affirming spaces, (2) characteristics of mental health provision, (3) healing journey process, and (4) community awareness and familiarity with services. Engaging with mental health providers of similar sexual and gender identities, alongside other identity groups (e.g., race, religion), enhanced therapeutic rapport through mutual connection and understanding. Counselors who adopted intersectional understandings in their practice and recognized the amplified barriers that SGM experience when accessing mental health services related to IPV may develop a stronger connection with service users. Healing was contextualized as positive when self-development was prioritized and when accessing individual counseling (rather than as a couple). Lastly, a facilitator to mental health service provision was community awareness and the integration of SGM-specific services. To increase the efficacy of mental health service provision and to promote the healing of SGM experiencing IPV, additional funding is needed to increase diversification of providers in the field and training in anti-oppressive frameworks within provider education curricula.

**Table:** Implications for Practice, Policy, and Research.

• Given the overall positive experiences of SGM with mental health supports in the context of IPV, governments and policymakers should prioritize resource allocation toward this sector to expand and diversify available services and reduce cost, waitlists, and transportation barriers to improve accessibility • Not only should the number of mental health supports be increased, but mental health providers and organizations should be required to undertake mandatory training modules to ensure providers are more attuned and sensitive to SGM individuals seeking help for IPV and to reduce IPV minimizations, generalizations, and stigmatization • To increase representation and SGM-affirming mental health supports, policies should be developed that increase scholarships and educational opportunities for SGM individuals to obtain relevant experience to become mental health professionals; by doing so, more providers with similar identities can be employed in this sector to increase accessibility for SGM clients experiencing IPV. This point also extends to individuals identifying with other marginalized communities (e.g., disability) to ensure that full representation is available within the mental health sector • Changes to mandatory reporting laws should be explored due to (a) the barriers posed to SGM IPV help-seeking and (b) the disproportionate impacts mandatory reporting laws incur on marginalized groups • Mental health support groups should be led by trained facilitators with additional resources to limit re-traumatization and to promote the navigation of IPV experiences • IPV bidirectionality is often misunderstood within the context of SGM relationships; couples counselors must ensure that bidirectionality of abuse is considered without blaming, minimizing, or stigmatizing experiences of IPV • Policies should be put into place that increase advertisements and outreach within different educational settings (e.g., high school) to ultimately increase awareness of available mental health supports for SGM experiencing IPV • Researchers in the field of SGM IPV and mental health should evaluate and refine existing IPV interventions (e.g., cognitive behavioral therapy) to determine the most suitable approaches in supporting SGM experiencing mental health difficulties in the context of IPV • More research is needed to illuminate how SGM experiences with mental health supports vary across intersecting identities; most studies are comprised of small, stratified groups, resulting in inadequate sample sizes to draw appropriate conclusions

*Note*. IPV = intimate partner violence and abuse; SGM = sexual and gender minorities.

## Supplemental Material

sj-docx-1-tva-10.1177_15248380251355918 – Supplemental material for Mental Health Supports for Sexual and Gender Minorities Who Experience Intimate Partner Violence and Abuse: A Systematic Review of North American Literature Supplemental material, sj-docx-1-tva-10.1177_15248380251355918 for Mental Health Supports for Sexual and Gender Minorities Who Experience Intimate Partner Violence and Abuse: A Systematic Review of North American Literature by Stefan Kurbatfinski, Katherine Maurer, Jessica Whitehead, Noah Ulicki, Lee Hodge, Martin Morris, René Peltekian, Richard Henry and Zack Marshall in Trauma, Violence, & Abuse

sj-docx-2-tva-10.1177_15248380251355918 – Supplemental material for Mental Health Supports for Sexual and Gender Minorities Who Experience Intimate Partner Violence and Abuse: A Systematic Review of North American Literature Supplemental material, sj-docx-2-tva-10.1177_15248380251355918 for Mental Health Supports for Sexual and Gender Minorities Who Experience Intimate Partner Violence and Abuse: A Systematic Review of North American Literature by Stefan Kurbatfinski, Katherine Maurer, Jessica Whitehead, Noah Ulicki, Lee Hodge, Martin Morris, René Peltekian, Richard Henry and Zack Marshall in Trauma, Violence, & Abuse

sj-docx-3-tva-10.1177_15248380251355918 – Supplemental material for Mental Health Supports for Sexual and Gender Minorities Who Experience Intimate Partner Violence and Abuse: A Systematic Review of North American Literature Supplemental material, sj-docx-3-tva-10.1177_15248380251355918 for Mental Health Supports for Sexual and Gender Minorities Who Experience Intimate Partner Violence and Abuse: A Systematic Review of North American Literature by Stefan Kurbatfinski, Katherine Maurer, Jessica Whitehead, Noah Ulicki, Lee Hodge, Martin Morris, René Peltekian, Richard Henry and Zack Marshall in Trauma, Violence, & Abuse
