# Supplementary material for: Mental Health Supports for Sexual and Gender Minorities Who Experience Intimate Partner Violence and Abuse: A Systematic Review of North American Literature
Source: Trauma Violence Abuse. 2025 Sep 20;27(4):1533–49. doi: 10.1177/15248380251355918 (PMC13578669; doi:10.1177/15248380251355918)
Supplement: Supplementary file 1 [file sj-docx-1-tva-10.1177_15248380251355918.docx]

**Supplementary Materials 1: Sample Medline Search Strategy**

1. exp "Sexual and Gender Minorities"/

2. exp Homosexuality/

3. exp Bisexuality/

4. exp Transsexualism/

5. exp Sex Reassignment Procedures/

6. exp "Health Services for Transgender Persons"/

7. (lgbt* or glbt* or "lgb+" or gay or bisexual* or homosexual* or pansexual* or "same sex" or two-spirit* or lesbian* or transsexual* or trans or transgender* or intersex* or queer or genderqueer or "gender fluid" or questioning or nonconforming or non-conforming or msm or mesm or "men who have sex with men" or "men having sex with men" or "women who have sex with women" or "non-hetero*" or non-monosexual* or nonbinary or non-binary or ((gender or sexual) adj1 (divers* or minorit* or identit*)) or "sexual orientation").mp.

8. "Health Services for Transgender Persons"/

9. or/1-8

10. exp Intimate Partner Violence/

11. Sex Offenses/ or Rape/

12. ((violence adj3 ("coercive controlling" or "intimate partner" or "situational couple" or dating or domestic)) or (partner adj3 (control or power)) or ((rape or violence or abuse) adj5 (marital or spouse* or spousal or partner* or dating or family)) or ("intimate terrorism" or "mutual violent control") or IPV).mp.

13. ((sex* adj1 (violence or violent or assault* or coerc* or force* or (against adj3 will))) or rape?).mp.

14. or/10-13

15. 9 and 14

16. ("friend? with benefits" or "hook* up?" or hookup? or ((casual or recreational or "emotionally uncommitted" or anonymous or informal or transactional) adj1 sex*) or ((unfamiliar or multiple or pre-marital or casual) adj1 partner?) or (fuck adj (budd* or friend? or boy?)) or "one night stand?" or "no strings" or "booty call?" or "netflix and chill").mp.

17. (violen* or victim* or surviv* or assault* or coerc* or force? or incapacitat* or abuse? or abusive* or batter* or aggress* or microaggress* or non-consensual* or "without consent").mp.

18. Violence/ or exp Physical Abuse/ or Aggression/

19. 17 or 18

20. 9 and 16 and 19

21. 15 or 20
