# Supplementary material for: Mental Health Supports for Sexual and Gender Minorities Who Experience Intimate Partner Violence and Abuse: A Systematic Review of North American Literature
Source: Trauma Violence Abuse. 2025 Sep 20;27(4):1533–49. doi: 10.1177/15248380251355918 (PMC13578669; doi:10.1177/15248380251355918)
Supplement: Supplementary file 2 [file sj-docx-2-tva-10.1177_15248380251355918.docx]

**Supplementary Materials 2:** Study information and main findings from included studies (N=34).

| **Study** | **Objective** | **Study Design & Sampling Method** | **Sample Size (n)** | **Mean, Median, or Ranged Age and Standard Deviation (years)** | **Sex, gender, and sexual orientation** | **Race/Ethnicity** | **Income Level** | **Educational Level** |
| --- | --- | --- | --- | --- | --- | --- | --- | --- |
| **Anderson & Overby, 2020, United States** | “to understand the unique barriers that service providers, who are also survivors of sexual violence, may experience and how it informs their work with survivors” ^(p1564)^ | Qualitative, grounded theory and feminist  Non-probability (quota, purposive, snowball sampling) | 19 | -36 | -Mostly females; 88% trans, and 12% non-binary service providers | -64% white non‐Hispanic  -36% non‐white (8% Latina; 8% African American, 8% American Indian; and 12% bi‐racial) | N/A | -Attended or completed college (76%)  -Attending college (16%)  -Completing graduate degrees (16%) |
| **Bornstein et al., 2006, United States** | “to understand community perspectives on domestic violence (DV) in lesbian, bisexual and trans (LBT) communities, and to assess access to and cultural appropriateness of DV services for LBT survivors” ^(p160)^ | Qualitative, participatory action research  Non-probability (purposive sampling) | 22 | -34 | -76.9% females, 23% trans  -69.2% lesbian, 30.7% bisexual | -81.8% white, 4.5%  Chicana, 13.6% Native American | -23.8% <$10,000, 47.6%  <$20,000 | N/A |
| **Brandt and Stults, 2024, United States** | “to qualitatively examine the experiences of coping with IPV victimization in a diverse sample of young sexual minority men (YSMM) living in the Greater New York region of the US” ^(p175)^ | Qualitative  Non-probability (quota, purposive sampling) | 25 | -26.4 (±1.01) | -80% gay, 16% bisexual, 4% queer | -4% Asian/Pacific Islander  -60% Black  -8% Latino  -16% multiracial  -12% white | -Perceived socioeconomic status: lower 17 (68%), middle 8 (32%), upper 0 (0%) | -24% high School  -28% some college  -32% college  -16% graduate school 4 |
| **Coston, 2019, United States** | to highlight "the voices of 38 queer and trans people of color (QTPOC)- identified U.S. Southerners who have experienced sexual violence and came together across three focus groups to detail their interactions with advocates and other professionals and explore posttraumatic needs" ^(p35)^ | Qualitative, focus groups  Non-probability  (quota, convenience, snowball, purposive sampling) | 38 | N/A | -Lesbian, gay, bisexual, trans, queer | -Mostly racialized individuals | N/A | N/A |
| **Coston, 2020, United States** | to examine “correlates of postvictimization health care utilization that stem from three major perspectives: the epidemiological lifecourse perspective (e.g. age), the importance of intersectionality (e.g. other identity and social location-based inequalities), and sexual orientation-based stigma (e.g. minority stress)” ^(pgs349-350)^ | Quantitative, cross-sectional  Probability  (Random sampling) | -1,027 | N/A | Self-identified and behaviourally bisexual or heterosexual | N/A | N/A | N/A |
| **de Heer et al., 2024, United States** | “to determine if the disclosure experience was helpful or harmful to the victim by directly interviewing individuals who identify as LGBTQA+ and experienced unwanted sexual contact” ^(p4)^ | Qualitative, queer and feminist approaches  Non-probability (Quota, purposive sampling) | 15 | -12+ | -33% lesbian, 27% queer women, 14% bisexual women, 14%  queer gender nonconforming, 6% queer transgender, 6% queer man  -73% women | N/A | N/A | N/A |
| **Donne et al., 2018, United States** | to fill "the gap in the literature and better understanding how men, both straight and gay as well as cisgender and transgender, conceptualize, understand, and seek help related to sexual violence" ^(p189)^ | Mixed-methods, survey, in-depth interviews  Non-probability  (quota, purposive sampling) | 188,  (19 interviews and 13 individuals in focus groups) | -32 (21-47) interviews  -32 (21- 47) focus groups | -84% assigned male sex at birth, 6% female, 11% declined to respond  -78% male, 4% transgender female to male, 2% transgender male to female, 16% agender, gender fluid, gender queer, or other, 1% female  -60% gay or lesbian, 16% bisexual, 11% straight, 2% asexual or other | -Racially diverse; 31% white, 29% Black or African American, 13% Latino/Hispanic, 6% Asian or Pacific Islander, 15% multiracial or other | N/A | -Majority undergraduate degree (~60%) |
| **Drouillard and Foster, 2024, Canada** | “to explore the  experiences of Two-Spirit, lesbian, gay, bisexual, transgender, queer, intersex, asexual,  and other sexual and gender diverse (2S/LGBTQIA+) people with IPV in Ontario  and how the COVID-19 pandemic affected their IPV experiences” ^(p1)^ | Qualitative,  Non-probability (quota, convenience,  purposive sampling) | 20 | -18-49  -Mostly under 35 (n=18) | -11 cisgender woman, 5 cisgender man, 3 non-binary/genderfluid, 1 transgender (man)  -11 bisexual/pansexual, 5 lesbian/gay, 3 asexual, 2 demisexual | Racially mixed, 9 white/Caucasian/Jewish, 7 Asian/South Asian/Chinese, 2  Black/African-American, 2  mixed/Brown | N/A | -Undergraduate degree or  Higher (N=9)  College diploma (N=3)  Some college/university (N=7)  High school diploma (N=1) |
| **Everhart & Hunnicutt, 2013, United States** | to explore “the experiences of self-identified queer victims of intimate partner violence, their personal encounters with violence-response organizations, and the extent to which their gender/ sexual identity impacted their willingness to disclose their abuse" ^(p67)^ | Qualitative,  Non-probability  (quota, snowball, purposive, sampling) | 11 | -18+ | -Queer | N/A | N/A | N/A |
| **Freeland et al., 2018, United States** | to examine “the perceptions of coping strategies, including formal services and informal sources of support, available to gay and bisexual male victims of IPV in Atlanta" ^(p303)^ | Qualitative, narrative  Non-probability  (venue-based sampling) | 64 | -34.5 | -55 gay, 9 bisexual  -64 men | Predominantly racialized sample, including 44 Black/African American, 15  Caucasian/white, 1  Hispanic/Latino, 2  Asian/Pacific Islander, 2 Other | N/A | -College or more (N=20)  Some college or 2-year degree (N=30)  High school diploma or GED (N=12)  No high school diploma (N=2) |
| **Hardesty et al., 2011, United States** | to explore “the individual, interpersonal, and sociocultural characteristics of lesbian/bisexual mothers who did and did not seek help for IPV. We also sought to extend Liang et al.’s framework by exploring how heterosexism, felt stigma, and legal vulnerability relate to help seeking among lesbian/bisexual mothers” ^(p31)^ | Qualitative, help-seeking framework  Non-probability (quota sampling) | 24 | 32 (±9) | -19 out lesbians  -5 closeted lesbians, bisexuals, or undefined | -12 African American, 9 white, 3 Latina | N/A | -From some high school to a bachelor’s degree |
| **Hereth, 2021, United States** | to explore “experiences of cumulative victimization across the life course and barriers to help-seeking behaviors among a sample of 21 transgender women in Chicago” ^(p461)^ | Qualitative, life-course, narrative  Non-probability (convenience sampling) | 21 | 26.6 (±­3.4) | -Transgender woman, woman, female, genderqueer  -All participants assigned male at birth | -81% Black/African American or Black/African  American and multiracial, 5%  Latina, 14% white | N/A | N/A |
| **Jackson et al., 2017, United States** | “to use narratives of SMM who experienced sexual assault (1) to explore responses to sexual assault disclosure by disclosure recipients; (2) to identify emotions and behaviors experienced by the men in response to their disclosures and secondary victimization experiences from disclosure recipients; (3) to increase understanding of how discrimination influenced past and future decisions to disclose sexual assault and subsequent interactions around disclosure; and (4) to explore the men’s beliefs about how secondary victimization has influenced sexual assault recovery” ^(p276)^ | Qualitative, minority stress framework  Non-probability (purposive sampling) | 18 | 42.3 (±12.3) | -78% gay, 17% bisexual, 6% queer | -100% white, non-Hispanic | N/A | -Trade certification (N=1), some college, no degree (N=4), associate’s degree (N=1), bachelor’s degree (N=8), advanced degree (e.g., master’s, doctoral, law, medical) (N=8) |
| **Kurdyla et al., 2021, United States** | to explore "help-seeking attitudes and behaviors of a convenience sample of 92 transgender adults and 325 cisgender sexual minority adults in the United States" ^(p11046)^ | Quantitative, questionnaire  Non-probability (convenience, snowball sampling) | 92 | -2.87 (±1.39), whereby 2 = 20-29 years and 3 = 30-39 years | -69.6% nonbinary, 13% transgender woman, 17.4% transgender man  -13% heterosexual, 10.9% bisexual, 8.7% lesbian, 3.3% gay, 56.5% queer, 7.6% other | -5.4% African American, 3.3%  Asian American, 2.2% Native American, 87%  White, 10.9% Latino, 9.8% other | N/A | N/A |
| **Kurdyla, 2023, United States** | “to understand the barriers to disclosure transgender survivors face and how they overcome these barriers” ^(p473)^ | Qualitative, inductive narrative  Non-probability (quota, snowball sampling) | 9 | 19-57 | -1 man/transman, 1 transgender, 3 nonbinary, 1 agender, 1 bigender/transwoman, 1 transwoman, 1 transman | -6 white, 2 people of colour, 1 other/Mediterranean | N/A | N/A |
| **Lippy et al., 2020, United States** | to explore “how MR laws impact survivors’ help-seeking, the outcomes of their help-seeking, and whether their race, gender, and/or sexual orientation influenced their experiences” ^(p255)^ | Mixed-methods, cross-sectional and inductive content analysis, survey and intersectionality  Non-probability (purposive sampling) | 2462 | 31 | -87.6% women, 10.8% men, 1.6% trans or gender non-conforming  -84.3% heterosexual, 7.8% bisexual, 3.8% gay/lesbian, 4.1% sexually fluid | -57.1 % white/Caucasian, 15.9% Latino/a/Hispanic, 14%  Black/African American, 7.1%  Asian/Asian American, 5.9%  multi-racial/mixed race | N/A | N/A |
| **Merrill & Wolfe, 2000, United States** | to investigate “the experiences of battered gay and bisexual men” ^(p1)^ | Quantitative, cross-sectional, survey  Non-probability (purposive sampling) | 52 | <25 (8%)  26-35 (54%)  36-50  (35%)  >50 (2%) | -96% gay, 4% bisexual  -Men | -29% African American, 29% European American, 19% Latino, 12% Asian/Pacific Islander, 4% Native, 7% Other | -15% <$10,000, 8% $10,000-$15,000, 21% $15,001-$25,000, 29%  $25,001-$35,000, 12% $35,001-$50,000, 10% >$50,000 | -10% graduate or professional school, 46% bachelor’s degree, 44% high school or equivalent |
| **Ollen et al., 2017, United States** | “to explore sexual minority college students’ perceptions of the prevalence of DV and SA, risk and protective factors, and barriers to seeking help, using focus groups” ^(p112)^ | Qualitative, general qualitative  Non-probability (quota, purposive sampling) | 14 | 18-24 | -4 gay, 3 lesbian, 3 bisexual, 1 pansexual, 1 queer  -6 cisgender men, 8 cisgender women | -86% white | N/A | -13 undergraduate, 1  graduate |
| **Oswald et al., 2010, United States** | “to examine lesbian mothers’ counseling experiences in the context of IPV through secondary analysis of in-depth interview data” ^(p289)^ | Qualitative  Non-probability (quota, purposive sampling) | 24 | 39.5 (±8.9) | -22 lesbian, 2 bisexual | -12 Black, 9 white, 3 Latina | N/A | -Associate’s degree (median level of education) |
| **Oueis et al., 2024, Canada** | “to collect and analyze the experiences of  gay, bisexual, and other men who have sex with men (GBMSM) to better understand a range of coping behaviors, also reflecting barriers to help-seeking and appropriate resources” ^(p416)^ | Qualitative, exploratory  Non-probability (quota sampling) | 206 | 31.84 | -73.3% male, 13.6% trans male, 5.8%  genderqueer/genderfluid, 1.9% agendered, 5.3% non-binary  -62.6% gay, 20.4% bisexual, 13.1%  queer, 0.5%  aromantic/asexual, 2.4%  pansexual, 1% questioning | -71.8% Caucasian/white, 4.4% Hispanic or Latino, 0.5%  Black or African American, 2.9%  Indigenous, 6.8%  Asian or Pacific Islander, 1.5%  South Asian, 2.9%  Middle Eastern or West Asian, 9.2% identify with multiple ethnic backgrounds | -35.4% <$25,000, 23.8% $26,000 - $49,00, 24.8%  $50,000 - $99,000, 10.7%  $100,000-$199.000, 1.5%  >$200,000 | -2.9% less than high school, 22.3% high school diploma, 21.4% college diploma, 32.5% bachelor’s degree, 16% master’s degree, 4.9%  Doctoral or Professional Degree |
| **Renzetti, 1988, United States** | to develop “an understanding of homosexual partner abuse by examining the incidence, forms, and correlates or violence in lesbian relationships” ^(p381)^ | Quantitative, cross-sectional  Non-probability (quota sampling) | 100 | -26-35 (47%) | -100% lesbian | -Mostly white (96%) | N/A | N/A |
| **Roy et al., 2024, Canada** | To examine, “through an ecological lens, the different risk and protective factors related to IPV victimization in a sample of gay men in the province of Québec, Canada, who acknowledge that they have experienced IPV, and illustrate how those factors affect their experience of victimization” ^(p1947)^ | Qualitative, ecological perspective  Non-probability (quota, purposive sampling) | 23 | 43 (median age) | -21 cisgender man, 1 heterosexual, 1 questioning sexual orientation and gender | -Majority Québec-born, 2 Latin America born, 1  Southern Africa | -52% >$40,000 (CAD) a year | -16 university degree |
| **Scherzer, 1998, United States** | “to gather information regarding the reported frequency of abusive or violent behaviors in a sample of lesbian relationships in the San Francisco Bay Area, 2. to assess respondents’ attitudes and beliefs about abusive or violent behaviors, 3. to review respondents’ help-seeking experiences” ^(p34)^ | Quantitative, cross-sectional  Non-probability (non-random, quota, snowball, purposive sampling) | 256 | -29% 21-30, 47% 31-40, 20% 41-50 | -Lesbian women | -44% women of colour; 12% Asian and Pacific Islander, 2% Native women, 10% African descent, 10% Latinas, 11% multiracial | -Income level  evenly distributed, but 31% majority reporting $20,000-$29,999 | -At least some college education (>97%), whereby 44% and 32% completed college and advanced degrees, respectively |
| **Schilit et al., 1991, United States** | “to investigate whether there is a relationship between domestic violence in the family of origin and subsequent abusive lesbian relationships” ^(p73)^ | Quantitative, cross-sectional  Non-probability (purposive sampling) | 104 | -34.7 (22 to 52) | -Lesbian | 93% white, 2% Hispanic, 1% Black, 1% Native, 3% Other | -4% <$2,400, 9% $2,500-$7,000, 23% $7,100-$12,000, 16% $12,100-$17,000, 21% $17,100-$25,000, 17% $25,000-$32,000, 8% $32,100-$50,000, 2% >$50,000 | -Eighth grade to  Doctoral degree, with most reporting a degree beyond undergraduate level (41%) |
| **Shultz, 2019, United States** | to examine “a need for research into guiding practices for operating such groups, as well as research on the conversational themes that occur in these spaces in order to inform future practices for peer supporters and clinicians” ^(p297)^ | Qualitative, autoethnography, trans methodology  N/A | N/A | N/A | -Trans individuals | -White (author) | N/A | N/A |
| **Simpson & Helfrich, 2014, United States** | “to identify and describe the barriers to service among a marginalized group of women and to reveal their recommendations for improved service accessibility and outreach” ^(p446)^ | Qualitative, intersectionality  Non-probability (purposive, convenience, sampling) | 16 | -39 (26 to 53) | -13 Lesbian, 3 bisexual | -100% Black | -Low-income, average of $258  Per month | -1 associate degree, 8 some college, 3 vocational training programs, 4 did not complete high school |
| **Soares et al., 2024, Canada** | “to explore the experience of help-seeking among women who identify as LGBTQ, who live rurally and who experienced IPV” ^(p210)^ | Qualitative, intersectionality, interpretive description utilizing a case-study design  Non-probability (purposive sampling) | 4 | -26.75 (±5.25) | -Women (1 transgender, 3 cisgender) | N/A | -2 <$50,000, 2 $50,000-$99,000 (CAD) | -2 high school, 2 college/university |
| **Todahl et al., 2009, United States** | to examine “the relationship between community attitudes toward LGBTQ persons and associated community responses to LGBTQ sexual assault victims” ^(p952)^ | Mixed-methods, cross-sectional and grounded theory, survey  Non-probability (convenience, snowball, purposive sampling) | Survey (n=130) or focus group interviews (n=14) | 36 (±13.9) | -63.8% female, 30.8% male, 4.6% transgender/other, 0.8% no response  -23.0% gay, 20.0% lesbian, 18.5% bisexual, 18.5% heterosexual, 19.2% other/multiple categories, 0.8% no response | -76.2% white, 23.8% minority status | N/A | N/A |
| **Turell, 1999, United States** | to survey “regarding use of resources to stop same-sex relationship abuses” ^(p35)^ | Quantitative, cross-sectional, survey  Non-probability (quota, purposive sampling) | 499 | 37.5 (±10.5) | -46% men, 53% women, 1% male to female transgendered  -11% gay women, 43%  gay men, 5%  bisexual, 2%  heterosexual | -75% white, 8%  African Americans, 8% Latinos, 4% multi-or bi-ethnic, 3% Native Americans, 1% Asian | -Mean $37,500 (±$30,550, range: $0- $850,000) | N/A |
| **Turell & Cornell-Swanson, 2005, United States** | to examine “within group differences regarding type of support sought and its usefulness” ^(p71)^ | Quantitative, cross-sectional, survey  Non-probability (quota, purposive sampling) | 677 | 37.7 (±10.2) | -59% female, 40% male, 1% transgender  -44.7% lesbian, 10.8%  gay woman, 37%  gay man, 5.9%  bisexual, 1.5%  heterosexual | -76% Caucasian, 9%  Latino, 7% African American, 4.5% bi- or multi-ethnic, 3%  Native, 0.6% Asian | - Mean $34,782 (±$27,798, $0- $300,000) or median $30,000 | N/A |
| **Walsh et al., 2023, United States** | “to understand barriers to seeking post-sexual assault services for students of color and lesbian, gay, bisexual, transgender, queer, and questioning (LGBTQ+) students” ^(p1)^ | Qualitative  Non-probability (purposive sampling) | 29 | N/A | -75.0% cisgender women, 10.3% cisgender men, 13.8% trans, non-binary, or questioning  -41.4% straight or heterosexual, 34.5% queer, 24.1% lesbian, bisexual, pansexual, or questioning | -20.7% Asian, 17.2% Black, 10.3% Hispanic, 37.9% white/non-Hispanic, 13.8% other | N/A | -23 undergraduate students, 6 Graduate student |
| **Wang, 2011, United States** | “to provide an in-depth understanding of one lesbian’s experience with same-sex sexual violence in a rural context” ^(p168)^ | Qualitative, case study, feminism  Probability (simple random  sampling) | 1 | 50s | -Lesbian | -White | N/A | N/A |
| **Whitton et al., 2024, United States** | “to explore: (a) whether SGM-AFAB disclose or seek help for IPV they experience, and from whom; (b) barriers to disclosure and help-seeking; and (c) responses to SGM-AFAB’s IPV disclosure and help-seeking, including the type of response or support provided and its perceived helpfulness” ^(p1376)^ | Mixed-methods  Purposive and snowball sampling | 41 | 21.63 (18 to 32) | -68.29% cisgender female, 31.71% gender minority, 2.44% transgender, 4.89% gender nonconforming, 7.32% gender queer, 17.07% nonbinary  -29.27% monosexual, 29.27% gay or lesbian, 46.34% non-monosexual, 21.46% bisexual, 4.89% pansexual, 24.39% other, 19.51% queer, 2.44% unsure or questioning, 2.44% asexual | -31.71% Black, 34.15% Latinx, 29.27% white, 4.89% other | N/A | N/A |
| **Wright et al., 2023, Canada** | “to investigate youths’ connections to other sexual and gender minorities, their sense of community support, and experiences  of transphobic, biphobic, and homophobic discrimination, as  well as experiences of various kinds of dating violence” ^(p252)^ | Quantitative  Non-probability (purposive, quota sampling) | 292 | 18  (14-24), most under 18 | -52% woman/girl, 24% man/boy, 30% trans, 20% transmasculine, 21% cisgender, 27% non-binary, 6% agender, 12%  Genderqueer, 12%  Genderfluid, <5%  Two-Spirit, <5% Transfeminine, <5% other  -36% bisexual, 32% queer, 25%  Pansexual, 22% lesbian, 17% gay, 17% questioning, 12% demi-sexual, <5% Two-Spirit, <5% aromantic, 6% other | -86% white, 9% Indigenous, 5%  Asian, South Asian, Black,  Latin American, or Middle Eastern | N/A | N/A |

NOTE: Terms for race/ethnicity, income, and education were pulled directly from the included studies.
