# Supplementary material for: Mental Health Supports for Sexual and Gender Minorities Who Experience Intimate Partner Violence and Abuse: A Systematic Review of North American Literature
Source: Trauma Violence Abuse. 2025 Sep 20;27(4):1533–49. doi: 10.1177/15248380251355918 (PMC13578669; doi:10.1177/15248380251355918)
Supplement: Supplementary file 3 [file sj-docx-3-tva-10.1177_15248380251355918.docx]

**Supplementary Materials 3:** Critical Appraisal Results (N=34).

| **Qualitative Studies (n=25)** | | | | | | | | | | | | | | | | | | | | | | | | | |
| --- | --- | --- | --- | --- | --- | --- | --- | --- | --- | --- | --- | --- | --- | --- | --- | --- | --- | --- | --- | --- | --- | --- | --- | --- | --- |
|  | Anderson & Overby, 2020 | Bornstein et al., 2006 | Brandt & Stults, 2024 | Coston, 2019 | de Heer et al., 2024 | Donne et al., 2018 | Drouillard & Foster, 2024 | Everhart & Hunnicutt, 2013 | Freeland et al., 2018 | Hardesty et al., 2011 | Hereth, 2021 | Jackson et al., 2017* | Kurdyla, 2023 | Lippy et al., 2020* | Ollen et al., 2017 | Oswald et al., 2010 | Oueis et al., 2024 | Roy et al., 2024 | Shultz, 2020 | Simpson & Helfrich, 2014 | Soares et al., 2024 | Todahl et al., 2009* | Wang, 2011 | Walsh et al., 2024 | Whitton et al., 2024* |
| Is there congruity between the stated philosophical perspective and the research methodology? | ✓ | ✓ | ✓ | ✓ | ✓ | ✓ | ✓ | ✖ | ✓ | ✓ | ✓ | ✓ | ✓ | ✓ | ✓ | ✓ | ✓ | ✓ | ✓ | ✓ | ✓ | ✓ | ✓ | ✖ | ✓ |
| Is there congruity between the research methodology and the research question or objectives? | ✓ | ✓ | ✓ | ✓ | ✓ | ✓ | ✓ | ✓ | ✓ | ✓ | ✓ | ✓ | ✓ | ✓ | ✓ | ✓ | ✓ | ✓ | ✓ | ✓ | ✖ | ✓ | ✓ | ✓ | ✓ |
| Is there congruity between the research methodology and the methods used to collect data? | ✓ | ✓ | ✓ | ✓ | ✓ | ✓ | ✓ | ✓ | ✓ | ✓ | ✓ | ✓ | ✓ | ✓ | ✓ | ✓ | ✓ | ✓ | ✓ | ✓ | ✖ | ✓ | ✓ | ✓ | ✓ |
| Is there congruity between the research methodology and the representation and analysis of data? | ✓ | ✓ | ✓ | ✓ | ✓ | ✓ | ✓ | ✓ | ✓ | ✓ | ✓ | ✓ | ✓ | ✓ | ✓ | ✓ | ✓ | ✓ | ✓ | ✓ | ✖ | ✓ | ✓ | ✓ | ✖ |
| Is there congruity between the research methodology and the interpretation of results? | ✓ | ✓ | ✓ | ✓ | ✓ | ✓ | ✓ | ✓ | ✓ | ✓ | ✓ | ✓ | ✓ | ✓ | ✓ | ✓ | ✓ | ✓ | ✓ | ✓ | ✓ | ✓ | ✓ | ✓ | ✓ |
| Is there a statement locating the researcher culturally or theoretically? | ✓ | ✓ | ✖ | ✓ | ✓ | ✓ | ✖ | ✖ | ✖ | ✖ | ✖ | ✖ | ✓ | ✓ | ✖ | ✖ | ✖ | ✓ | ✓ | ✖ | ✓ | ✖ | ✖ | ✖ | ✓ |
| Is the influence of the researcher on the research, and vice- versa, addressed? | ✓ | ✖ | ✖ | ✖ | ✖ | ✖ | ✖ | ✖ | ✖ | ✖ | ✖ | ✖ | ✓ | ✓ | ✖ | ✖ | ✖ | ✓ | ✓ | ✖ | ✖ | ✖ | ✓ | ✓ | ✖ |
| Are participants, and their voices, adequately represented? | ✓ | ✓ | ✓ | ✓ | ✓ | ✓ | ✓ | ✓ | ✓ | ✓ | ✓ | ✓ | ✓ | ✓ | ✓ | ✓ | ✖ | ✓ | ✓ | ✓ | ✓ | ✓ | ✓ | ✓ | ✖ |
| Is the research ethical according to current criteria or, for recent studies, and is there evidence of ethical approval by an appropriate body? | ✖ | ✓ | ✓ | ✖ | ✖ | ✓ | ✓ | ✓ | ✓ | ✖ | ✓ | ✓ | ✓ | ✓ | ✓ | ✓ | ✓ | ✓ | ✓ | ✓ | ✓ | ✓ | ✓ | ✓ | ✓ |
| Do the conclusions drawn in the research report flow from the analysis, or interpretation, of the data? | ✓ | ✓ | ✓ | ✓ | ✓ | ✓ | ✓ | ✓ | ✓ | ✓ | ✓ | ✓ | ✓ | ✓ | ✓ | ✓ | ✓ | ✓ | ✓ | ✓ | ✓ | ✓ | ✓ | ✓ | ✓ |

| **Analytical Cross-Sectional Studies (n=13)** | | | | | | | | | | | | | |
| --- | --- | --- | --- | --- | --- | --- | --- | --- | --- | --- | --- | --- | --- |
|  | Coston, 2020 | Jackson et al., 2017* | Kurdyla et al., 2021 | Lippy et al., 2020* | Merrill & Wolfe, 2000 | Renzetti, 1988 | Scherzer, 1998 | Schilit et al., 1991 | Todahl et al., 2009* | Turell, 1999 | Turell & Cornell-Swanson, 2005 | Wright et al., 2023 | Whitton et al., 2024* |
| Were the criteria for inclusion in the sample clearly defined? | ✓ | ✓ | ✓ | ✓ | ✓ | ✓ | ✓ | ✖ | ✓ | ✓ | ✖ | ✓ | ✖ |
| Were the study subjects and the setting described in detail? | ✓ | ✓ | ✓ | ✓ | ✓ | ✓ | ✓ | ✓ | ✓ | ✓ | ✓ | ✓ | ✓ |
| Was the exposure measured in a valid and reliable way? | ✓ | ✓ | ✓ | ✓ | ✖ | ✓ | ✖ | ✓ | ✖ | ✖ | ✖ | ✓ | ✓ |
| Were objective, standard criteria used for measurement of the condition? | ✓ | ✓ | ✓ | ✓ | ✓ | ✓ | ✓ | ✓ | ✓ | ✓ | ✓ | ✓ | ✓ |
| Were confounding factors identified? | ✓ | N/A | ✓ | ✓ | ✖ | ✖ | ✓ | ✖ | ✓ | ✓ | ✓ | N/A | ✖ |
| Were strategies to deal with confounding factors stated? | ✓ | N/A | ✓ | ✖ | ✖ | ✖ | ✓ | ✖ | ✓ | ✓ | ✓ | N/A | ✓ |
| Were the outcomes measured in a valid and reliable way? | ✓ | ✓ | ✓ | ✓ | ✖ | ✓ | ✓ | ✓ | ✖ | ✓ | ✖ | ✓ | ✓ |
| Was appropriate statistical analysis used? | ✓ | ✓ | ✓ | ✓ | ✓ | ✓ | ✓ | ✓ | ✓ | ✓ | ✓ | ✓ | ✓ |

NOTE: Asterisk (*) signifies mixed-method study
